# Survey of current practices from an international task force for gynecological stereotactic ablative radiotherapy

**DOI:** 10.1186/s13014-020-1469-8

**Published:** 2020-01-30

**Authors:** E. Leung, A. Gladwish, A. Sahgal, S. S. Lo, C. A. Kunos, R. M. Lanciano, C. A. Mantz, M. Guckenberger, T. M. Zagar, N. A. Mayr, A. R. Chang, S. Jorcano, T. Biswas, A. Pontoriero, K. V. Albuquerque

**Affiliations:** 1 Department of Radiation Oncology, Odette Cancer Centre, Sunnybrook Health Sciences Centre, University of Toronto, Toronto, ON Canada; 2 Royal Victoria Hospital, Barrie, ON Canada; 3 Department of Radiation Oncology, University of Washington, Seattle, WA USA; 4 National Cancer Institute, Rockville, MD USA; 5 Delaware County Memorial Hospital/Philadelphia Cyberknife, Drexel Hill, PA USA; 6 21st Century Oncology, Fort Myers, FL USA; 7 University Hospital Zuerich, Zuerich, Switzerland; 8 Northeastern Radiation Oncology, Glen Falls, NY USA; 9 Soonchunhyang University Seoul Hospital, Seoul, South Korea; 10 Instituto Oncologico Teknon, Barcelona, Spain; 11 Case Western Reserve University School of Medicine, Cleveland, OH USA; 12 University of Messina, Messina, Italy; 13 University of Texas Southwestern Medical Center, Dallas, TX USA

**Keywords:** Stereotactic, Radiation, Gynecological, Consensus, SABR, SBRT

## Abstract

**Background:**

Stereotactic Ablative Radiotherapy (SABR) is an effective treatment that improves local control for many tumours. However, the role of SABR in gynecological cancers (GYN) has not been well-established. We hypothesize that there exists considerable variation in GYN-SABR practice and technique. The goal of this study is to describe clinical and technical factors in utilization of GYN-SABR among 11 experienced radiation oncologists.

**Materials and methods:**

A 63 question survey on GYN-SABR was sent to 11 radiation oncologists (5 countries) who have published original research, conducted trials or have an established program at their institutions. Responses were combined and analyzed at a central institution.

**Results:**

Most respondents indicated that salvage therapy (non-irradiated or re-irradiated field) for nodal (81%) and primary recurrent disease (91%) could be considered standard options for SABR in the setting of inability to administer brachytherapy. All other indications should be considered on clinical trials. Most would not offer SABR as a boost in primary treatment off-trial without absolute contraindications to brachytherapy. Multi-modality imaging is often (91%) used for planning including PET, CT contrast and MRI. There is a wide variation for OAR tolerances however small bowel is considered the dose-limiting structure for most experts (91%). Fractionation schedules range from 3 to 6 fractions for nodal/primary definitive and boost SABR.

**Conclusions:**

Although SABR has become increasingly standard in other oncology disease sites, there remains a wide variation in both clinical and technical factors when treating GYN cancers. Nodal and recurrent disease is considered a potential indication for SABR whereas other indications should be offered on clinical trials. This study summarizes SABR practices among GYN radiation oncologists while further studies are needed to establish consensus guidelines for GYN-SABR treatment.

## Background

Radiation plays an important role in the treatment of gynecological cancers. In both the definitive and adjuvant setting, local radiotherapy to pelvic primaries and lymph nodes have been shown to improve local control and outcome in different gynecological malignancies such as cervical, uterine and vulvovaginal tumours [1–6]. Brachytherapy has been a mainstay of gynecological (GYN) cancer treatments as the high conformality of this technique allows for high dose treatment to the central tumour while sparing nearby organs-at-risk [7]. Because of the effectiveness of brachytherapy and its importance in outcome in GYN cancer treatment, the role of stereotactic ablative radiotherapy (SABR) has not been well-studied.

SABR is well-known to be an effective radiation treatment technique for multiple tumour types and for various indications. Malignancies of the the lung, liver, brain, and the spine have well-established protocols and consensus in regard to treatment indications, technique and dose [8, 9]. SABR is defined as the delivery of ablative doses of radiation to localized targets sites using highly conformal techniques and is achieved by very robust immobilization, organ motion management, on-board imaging and advanced radiotherapy treatment planning.

A systematic review of SABR treatment for GYN cancers showed that this technique, although not standardly indicated, has been used for multiple GYN indications in a small number of single arm prospective clinical trials and several smaller retrospective series [10–12]. These include primary boost treatment for cervical and uterine cancers, adjuvant vaginal treatment and nodal or primary salvage treatment. The treatment has been generally well-tolerated with acceptable toxicities in the GYN setting [10]. However, it is apparent that clinical indications and techniques in these studies are vastly different and a standardized approach has not been adopted for GYN tumours worldwide. The goal of this study is to determine clinical and technological factors of GYN SABR among 11 radiation oncologists at 11 specialized SABR centres.

## Material and methods

A 63 question survey was sent to 11 radiation oncologists who practice and have published on GYN SABR in 5 different countries. Participating institutions included Department of Radiation Oncology, Sunnybrook Health Science Centre and Odette Cancer Centre, (OCC: Toronto, Ontario, Canada), National Cancer Institute, (NIH: Rockville, MD, USA), Delaware County Memorial Hospital/Philadelphia Cyberknife (PhilCyber: Drexel Hill, PA), twenty-first Century Oncology (21Century: Fort Myers, FL, USA), University Hospital Zuerich, (ZUR: Zuerich, Switzerland), Department of Radiation Oncology, Northeastern Radiation Oncology, (NE: Glen Falls, NY), Soonchunhyang University Seoul Hospital (SC: Seoul, Korea), Institut Oncologic Teknon, (TEK: Barcelona, Spain), Case Western Reserve University School of Medicine, (CASE: Cleveland, OH), Universita degli Studi di Messina, (MI: Messina, Italy), University of Texas Southwestern Medical Center, (UTSW: Dallas, TX). The survey included 63 questions in 5 parts. Topics of the questionnaire were pertaining to clinical and technological factors of GYN SABR technique. These included questions on the indications for GYN SABR, the eligibility criteria, pre-treatment investigation, treatment planning protocol, target delineation, dose targets and constraints and treatment response evaluation. Surveys were sent via e-mail and responses were collected and evaluated at a central institution (OCC). The questionnaire was completed during the period of January 2017 to March 2017. Responses were presented as percentages of agreement for each question among the 11 participants.

## Results

### Eligibility and indications

All participants completed the survey. The median number of GYN SABR patients treated in total per participant was 40 (5–125). SABR for GYN malignancies is used most commonly for recurrent disease (nodal or primary) in patients who have had prior radiation and/or have contraindications to brachytherapy or surgery (64–91%). Salvage pelvic and para-aortic nodal SABR was highly cited in this international survey as a common practice in patients with locally advanced gynecological cancers without prior radiation (82%).

SABR is not typically used as an alternative to brachytherapy but could be considered in the setting of clinical trials (64%). Primary SABR boost can be used when there is a contraindication to brachytherapy (73%). The indications of GYN SABR from the survey are summarized in Table 1.

**Table 1:** GYN SABR indications and number of respondents that offer this type of treatment

GYN SABR Indications	# of Respondents
Salvage Nodal	9
Salvage Pelvis/Primary	9
Cervical Boost	4
Endometrial Boost	4
Adjuvant Radiation	1
Vulva-Vaginal	2

Applying a size constraint for GYN SABR was common in setting of cervical/vaginal boost and nodal SABR (64%). Tumour size constraints varied between 5 and 8 cm or 90–600 cc. Nodal size limits varied from 3 to 5 cm or 125–600 cc. An age restriction is not common for SABR treatment in GYN cancers (91%) however some respondents felt that patients should generally have a performance status of 0–2 ECOG or KPS (64%).

It is recommended that pathological confirmation is obtained prior to starting SABR treatment (82%), however, histological subtype (eg. squamous cell carcinoma vs. adenocarcinoma) does not typically influence SABR treatment decision. (100%).

Contraindications to GYN SABR include brachytherapy eligibility (64%), tumour in close proximity to small bowel (46%), and previous high dose radiation/brachytherapy to the target site (46%).

### Imaging and treatment

Multi-modality imaging for staging and tumour assessment is typically performed prior to SABR treatment, including computerized tomography (CT) (82%), positron emission tomography (PET-CT) (82%), magnetic resonance imaging (MRI) (91%). For planning CT, slice thickness of 2 mm or less is used (82%). Most common organ motion strategies include fiducial markers (82%) and bladder protocol (82%). Intravenous contrast is often used with CT simulations (73%) with no routine use of premedication. Common treatment delivery techniques include VMAT (73%) and cyberknife (64%), with some centres using both techniques. Photon energy range between 6 MV to 18 MV.

Immobilization devices used include leg immobilizers (36%) and full body fix vaclock (64%). Implanted fiducials and kV x-rays are common for image guidance (64%). Other techniques include cone-beam CT (45%), robotic tracking (45%) and daily orthogonal KV x-rays (36%), with some centres using multiple techniques. Daily pre-treatment imaging and online correction is used. Rotation correction errors of > 1–3 mm are recommended (91%), with repeat imaging after couch adjustment (73%). Interfraction monitoring with fiducials is also recommended but post-treatment imaging is rare (45%). Checking pre-treatment imaging is important before each fraction (82%).

When chemotherapy is indicated, most respondents treat sequentially (73%), with a break of at least 1–3 weeks. The remaining respondents do not typically treat with SABR around the the time of systemic therapy.

### Target definitions and OARs

When considering GYN SABR for recurrent nodal disease, the clinical target volume (CTV) is considered to be the gross nodal disease with an expansion (73%) whereas 9% considers the CTV to be the gross tumour volume (GTV) only and another 9% considers the CTV to be the GTV plus the nodal region at risk. A planning target volume (PTV) expansion of 2–7 mm is typically applied. For the CTV of recurrent primary disease (eg. cervix), most treatment consider this to be the GTV or an expansion on the GTV (36–46%). Of the 8 participants that have used SABR as a boost treatment, 3 defined the target as the GTV + cervix + grey zones while the rest only treated the GTV +/− expansion.

PTV is often trimmed from organs at risk (OARs) to avoid overlap (82%), however a safety margin on OARs, or planning-at-risk-volumes (PRVs), is not typically used. Coverage of PTV is most commonly 95%. For CTV, 95–100% and GTV coverage is commonly 100%. Most common parameter used from planning optimization is conformity indices (82%).

Target dose prescription is prescribed to the periphery of the target (73%). Maximum hotspots can range between 105 and 200%. The isodose line covering the periphery can range from 65 to 90%.

### Dose fractionation and OAR constraints

Different fractionation doses and schedules are in practice for definitive SABR for recurrent disease and boost SABR treatment after external beam radiation. Fractionation regimens typically are between 3 and 5 fractions. For recurrent nodal SABR, median dose is 36 Gy EQD2 _(α/β = 10)_ (26.8–71.2). For recurrent primary SABR, median 40.4 EQD2 _(α/β = 10)_ (27–71.2). For boost SABR after standard pelvic radiation, Median 36.75 EQD2 _(α/β = 10)_ (15.6–60). Fractionation schedules for different centres participating in this study are summarized in Table 2. Typically, treatment is not on consecutive days and a minimum 1-day gap is kept between fractions (73%).

**Table 2:** SABR Dose fractionations for different centres for 2 indications: 1) nodal recurrences, 2) recurrent primary tumours and 3) primary boost

SABR Treatment	Centre 1	Centre 2	Centre 3	Centre 4	Centre 5	Centre 6	Centre 7	Centre 8	Centre 9	Centre 10	Centre 11
Nodal Recurrence	8 Gy × 3 5 Gy × 5	5–8 Gy × 5	8 Gy × 3	5–8 Gy × 3–5	5–7 Gy × 3	8–10 Gy × 5	11–13 Gy × 3 7.6–8 Gy × 5	4 Gy × 3 6 Gy × 5	10 Gy × 3	5–6 Gy × 5	6–8 Gy × 5
Recurrent Primary Tumor	Does not treat	5–8 Gy × 5	8 Gy × 3	5–8 Gy × 3–5	5 Gy × 4 5 Gy × 5	8–10 Gy × 5	11–13 Gy × 3 7.6–8 Gy × 5	4 Gy × 3 6 Gy × 5	10 Gy × 3	5–6 Gy × 5	6–8 Gy × 5
Primary Boost	NA	5 Gy × 2–3	NA	5.5 Gy × 5 (median)	5–7 Gy × 3	8 Gy × 5	5–8 Gy × 3–5	NA	7 Gy × 4	7 Gy × 4	5.5–6 Gy × 5

OARs most commonly limited to a dose constraint in GYN SABR include rectum, bladder, sigmoid, bowel, spinal cord and kidney with most recommending small bowel as the dose limiting organ (91%). A summary of dose constraints used for GYN SABR are presented in Table 3.

**Table 3:** OAR constraints from institutional policies of the 11 respondents of the survey. For 3 and 5 fraction SABR treatments

Dose constraints	Constraint for 3 fxs		Constraint for 5 fxs
OAR	De novo	De novo	reRT
Rectum	Dmax < 18–35 Gy V60–90% < 10 - 24Gy	Dmax< 37.5 - 38Gy D2cc < 38Gy V25Gy < =10 cc	Dmax< 25 - 30Gy D2cc < 32Gy
Bladder	Dmax: < 20–40 Gy V60–90% < 12- 24Gy	Dmax< 37.5–42 Gy V35Gy < 5%	Dmax< 25 - 30Gy D2cc < 36Gy
Sigmoid	Dmax < 20–33 Gy	Dmax < 39 - 40Gy V25Gy < 20 cc	D2cc < 32Gy Dmax< 25Gy
Large Bowel	Dmax < 9–33 Gy	Dmax< 38–40 Gy D2cc < 34Gy V25Gy < 20 cc	Dmax< 25 - 30Gy D2cc < 32Gy
Small Bowel	Dmax: 9–33 Gy < 1 cc > 24Gy V50% < 10Gy	Dmax< 35–39 Gy V25Gy < 5 cc	Dmax< 15 - 25Gy max D2cc < 20Gy
Skin	V60–95% < 12 - 24Gy		
Kidney	D200cc <16Gy Dmax <15Gy V50–90% < 10-14Gy	D200cc < 17.5Gy mean < 10–11 Gy V18Gy < 35%	mean < 10Gy

### Follow-up and response

SABR follow-up done in clinic every 3, 6 or 12 months with gynecological exam and investigations, including MRI, PET and CT contrast. Imaging is typically done 2–4 months after treatment to limit false positives from radiation changes. Local control is assessed through RECIST criteria or negative PET scan. Post-treatment biopsy is rare.

## Discussion

To our knowledge, this is the first study summarizing the practice and technological considerations of GYN SABR among experts that practice and have published on this technique [8, 10–21]. It is not intended to serve as a consensus guideline. GYN SABR is routinely used as a standard treatment option in a limited number of centres with some indications only recommended on clinical trial. Most common indications include salvage treatment to the pelvis with most experts agreeing that SABR should not be a replacement for brachytherapy. Brachytherapy is known to be an effective treatment essential to definitive treatment of locally advanced gynecological disease and should not be substituted by an external beam technique, including SABR. SABR techniques are observed to vary among experts, particularly dose fractionation and constraints, however, most policies are consistent in imaging and immobilization protocols.

**Fig. 1:**
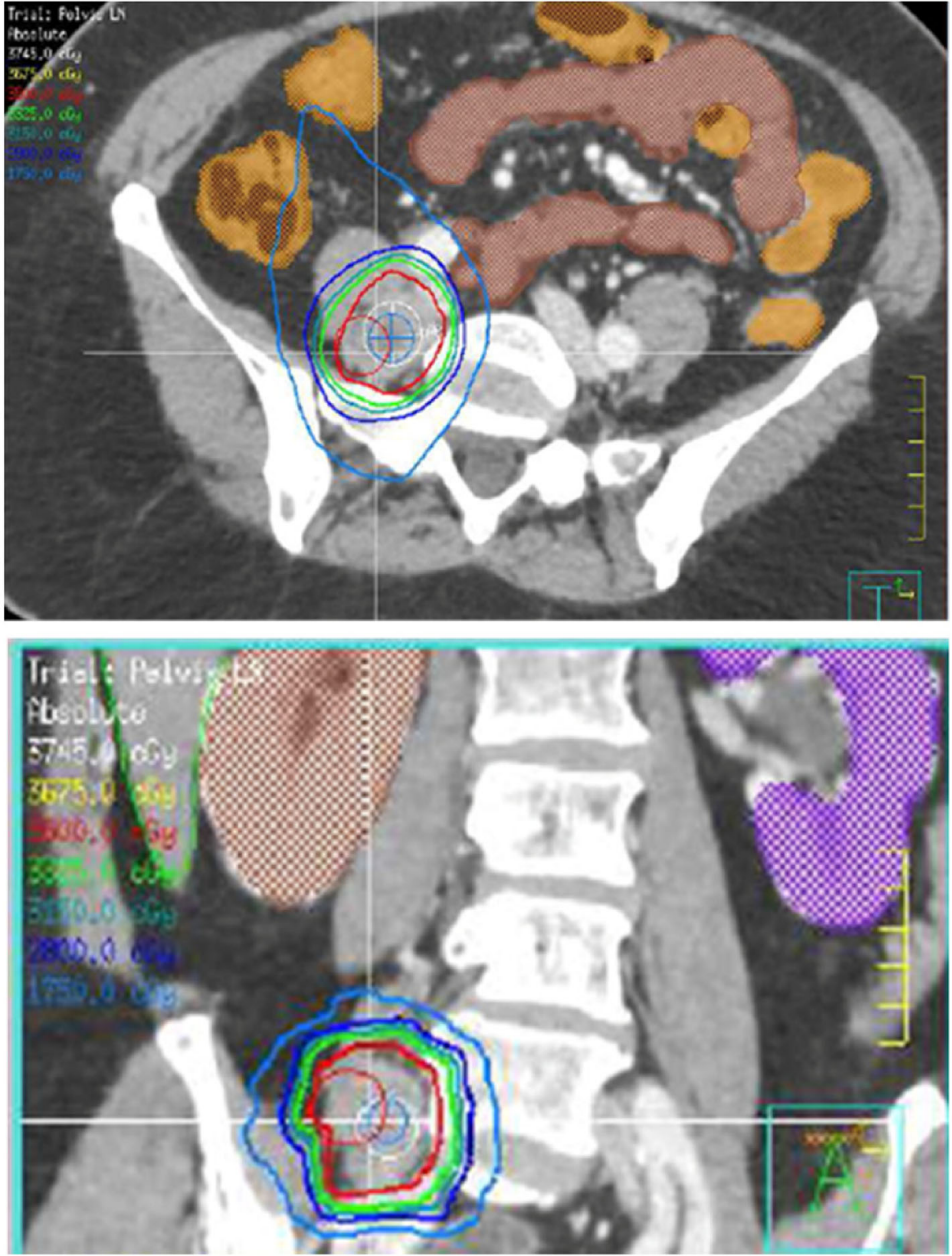
GYN SABR of recurrent nodal disease from endometrial cancer Nodal recurrence is bordering previous treatment field. Patient had previous FIGO IB, Grade 2 endometrioid adenocarcinoma with multifocal LVSI (no nodes removed) treated with pelvic radiation 4 years prior to SABR treatment. Prescription dose 35 Gy in 5 fractions

As GYN SABR is becoming increasingly common in practice, aligning international protocols may help guide the establishment of this technique as one of the standard options for certain clinical indications. In the systematic review by Mendez et al., six major clinical indications of GYN SABR were found in the published literature [10]. These included SABR as treatment for cervical cancer boost, endometrial cancer boost, salvage nodal treatment, pelvic recurrences, adjuvant treatment and vulvo-vaginal treatment. In this survey, salvage treatment for nodes and pelvic recurrences (when brachytherapy was contraindicated) were found to be the most common standard indications for SABR. Previously published reports have found this to be an effective treatment with local control rates above 80% [14, 22, 23]. Although some cases of isolated nodal recurrences in previous non-irradiated fields could be potentially salvaged with definitive chemoradiation treatment (and this is still the traditional approach), salvage nodal SABR is becoming more common in re-irradiation and oligometastatic scenarios (Fig. 1). The respondents to the survey (being heavily represented by an International affiliation) also cited salvage nodal SABR as an acceptable approach. The incorporation of isolated nodal SABR with added systemic therapy into current practice seems logical as it would allow rapid initiation of systemic therapy following SABR, as compared to the traditional approach of large field nodal salvage radiation which can take weeks to complete.

It should be noted that the survey results don’t reflect the more standard North American approach of salvage extended field nodal radiation and more trials are needed in this area before such practice is changed. With the emergence of SABR treatment for oligometastatic disease, there is increasing interest in this strategy for GYN tumours [24]. Several non-randomized studies and systematic reviews have shown a potential benefit of SABR treatment for oligometastatic disease in other solid tumours, such as breast, colorectal, lung and prostate. These studies have generally demonstrated that SABR for oligometastases yields high local control and may confer improved disease survival [25–28]. SABR-COMET was a phase II randomization between SABR and standard of care palliative radiation. In 99 patients, the study showed an improvement in survival within the SABR arm, but the study mostly included other solid tumour types as there were only two patients in SABR-COMET that had gynecological cancers. Median survival in the SABR arm was 41 months as compared with 28 months in the standard of care arm. However, it should be noted that only 3 patients in the SABR arm had treatment to nodal sites. Given the results of this survey and the existing literature in nodal GYN SABR, there is interest in prospectively studying oligometastases in solely gynecological cancers [12, 14, 29].

Some would consider boost treatment with SABR, however, most agree that this is only an option when there is a contraindication to brachytherapy. Although SABR can deliver high conformal doses of radiation to the target, its ability to deliver high central pelvic doses is still inferior to brachytherapy with current technologies and this can lead to suboptimal outcomes [7, 30]. In the Mendez systematic review, it was found that vulvar SABR was not common and has not been shown to be effective, and this was consistent with only 2 respondents treating vulvar SABR off of clinical trials. Adjuvant radiation using SABR instead of vaginal vault brachytherapy was only used by 1 respondent.

Imaging and treatment protocols were found to vary amongst the participating centres. GYN radiation oncologists have generally been late to adopt conformal techniques due to concerns of significant degrees of organ motion such as the cervix which has been shown to move as much as 4 cm in the anterior-posterior directions [31]. Therefore, most institutions generally employ strategies to address organ and tumour motion for SABR treatment including fidicual markers and organ-filling protocols. Multi-modality imaging was found to be essential due to the fact that different GYN diseases are best delineated with have different imaging modalities. For instance, soft tissue pelvic tumours are optimally delineated on MRI and while nodal disease are best appreciated on PET. With the recent introduction of MRI-based LINAC treatment systems, there is significant potential for this technology to play a role in GYN SABR [32]. With the challenge of organ motion in GYN tumours, MRI may help address this issue through on-line and real-time imaging.

CTV definitions were generally similar among participants with some using brachytherapy contouring guidelines to define targets in the central pelvis. Dose fractionation varied for different indications and varied among participants with most using 3–5 fractions for SABR treatment. There was also a wide variation of OAR constraints used with most treatment target constraints being similar in both the de novo and re-irradiation setting. These constraints reflect the institutional practices of the respondents and are not intended to serve as a consensus guideline for GYN SABR planning. Well-researched dose constraints that are not specific to GYN SABR include the AAPM-101 report and the more recent updated UK Consensus [33, 34]. Further research into toxicities and OAR tolerance specific to GYN SABR is needed .

## Conclusions

GYN SABR is commonly used in specialized centres around the world. Although SABR can deliver high-dose conformal radiation to targets, most experts agree that it is only used as a definitive boost treatment when brachytherapy is contraindicated. SABR for nodal and pelvic tumour salvage is becoming one of the common treatments and dose-fractionation and OAR tolerances vary among different centres. Future collaborative studies and clinic trials are warranted to better define the role and to standardize the practice of SABR for gynecological malignancies.
